# A Rare Case of Prosopagnosia Related to Intracranial Hemorrhage

**DOI:** 10.7759/cureus.45128

**Published:** 2023-09-12

**Authors:** Peyton Lampley, Michael D Saggio, Madeline L Boulet, Laurence Dubensky, Erin M Marra

**Affiliations:** 1 Emergency Medicine, HCA Florida Aventura Hospital, Aventura, USA; 2 Neurology, Larkin Community Hospital, Miami, USA

**Keywords:** facial dysmorphia, posterior circulation stroke, posterior cerebral artery stroke, intracranial hemorrhage (ich), prosopagnosia

## Abstract

Prosopagnosia describes the inability to recognize others by their faces, which may be hereditary or acquired. Acquired cases result from intracranial lesions such as intracranial hemorrhage or ischemia. This case demonstrates acquired prosopagnosia secondary to an intracranial hemorrhage and thus exemplifies the importance of early symptom recognition for appropriate diagnosis and management. A 58-year-old female presented to the emergency department with a chief complaint of the worst headache of her life along with nausea and vomiting. She also reported that she was unable to recognize her children in photos and although she knew her husband was with her, she did not recognize his face. Physical examination revealed no focal motor deficits. Computed tomography angiography of the brain revealed intracerebral hemorrhage of the right occipital lobe.

Acquired prosopagnosia can be the only presenting symptom of intracranial pathology. It is most commonly caused by intracranial hemorrhage, as shown in this case report. This demonstrates a unique symptom of posterior circulation strokes that are commonly misdiagnosed in the emergency department.

## Introduction

Prosopagnosia, often known as face blindness, is characterized by the inability to recognize others by their faces [1]. The word comes from the Greek word *prosopon*, meaning face, and *agnosia*, meaning lack of knowledge [1]. Prosopagnosia was first described in 1947 by Joachim Bodamer in a paper outlining two patients with difficulties in facial recognition. This facial blindness can include family members, pets, and even one’s own face in the mirror. Prosopagnosia may be acquired or hereditary. The vast majority of documented cases are hereditary with a possible autosomal dominant or polygenic inheritance pattern [2]. Acquired cases result from intracranial lesions such as intracranial hemorrhage or ischemic insults [3]. In this case, a patient presented to the emergency department with a newly acquired prosopagnosia as her only neurologic deficit secondary to an intracranial hemorrhage. This case exemplifies the importance of understanding acquired prosopagnosia as a symptom of intracranial pathology.

## Case presentation

A 58-year-old female with a past medical history of migraines and anxiety presented to the emergency department with a chief complaint of headache. The patient reported that three days prior to arrival, she had a strong, right-sided occipital headache that she described as the worst headache of her life and different from her normal migraines. The patient subsequently developed nausea and vomiting for the two days prior to arrival. About six hours prior to arrival to the emergency department, the patient reported that she noticed faces appeared strange to her. She reported that pictures of people she knew were unrecognizable. She described the faces as looking like “clowns” and being particularly distorted around the mouth. The patient reported that although she knew it was her husband at bedside, she was unable to recognize his face. The patient had no other neurologic symptoms.

**Figure 1:**
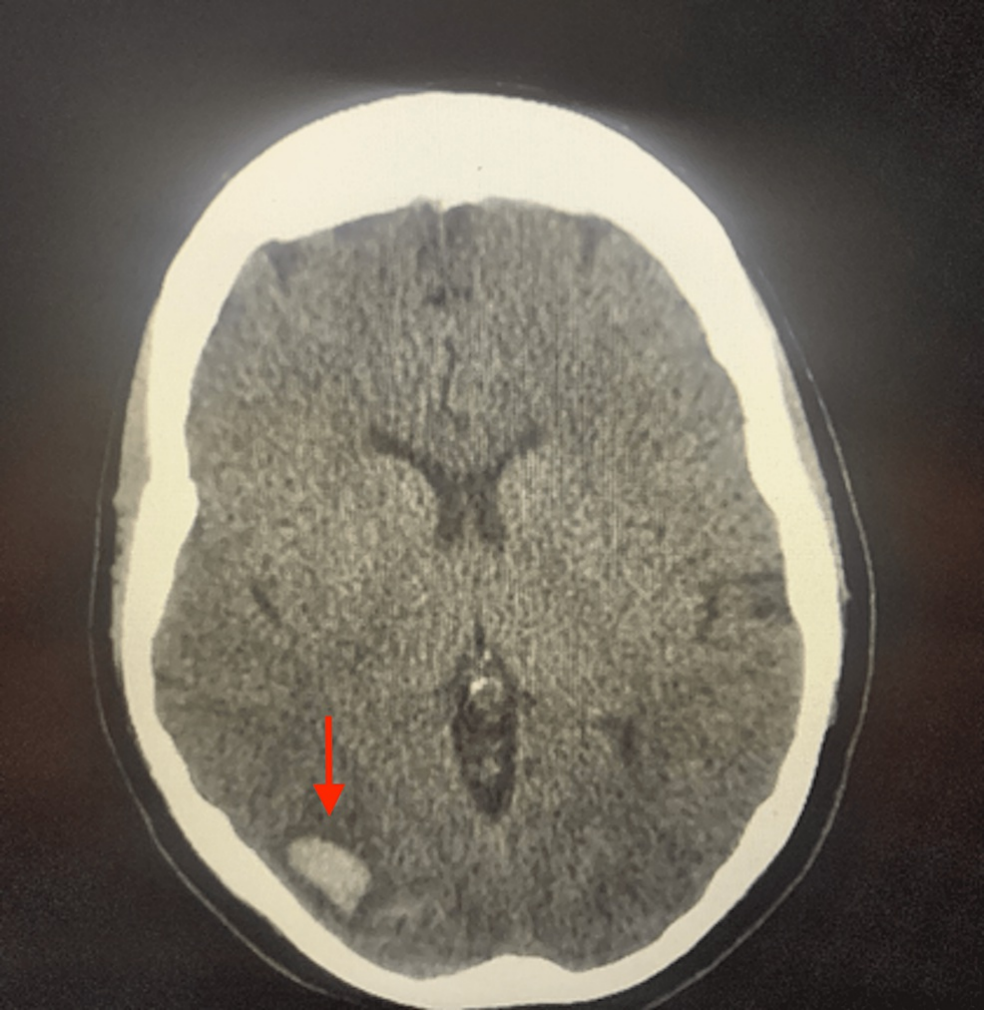
Transverse section from the initial brain CT without contrast done in the emergency department showing a right occipital hematoma measuring 1.1 x 1.8 x 1.6 cm (3.2 mL)

**Figure 2:**
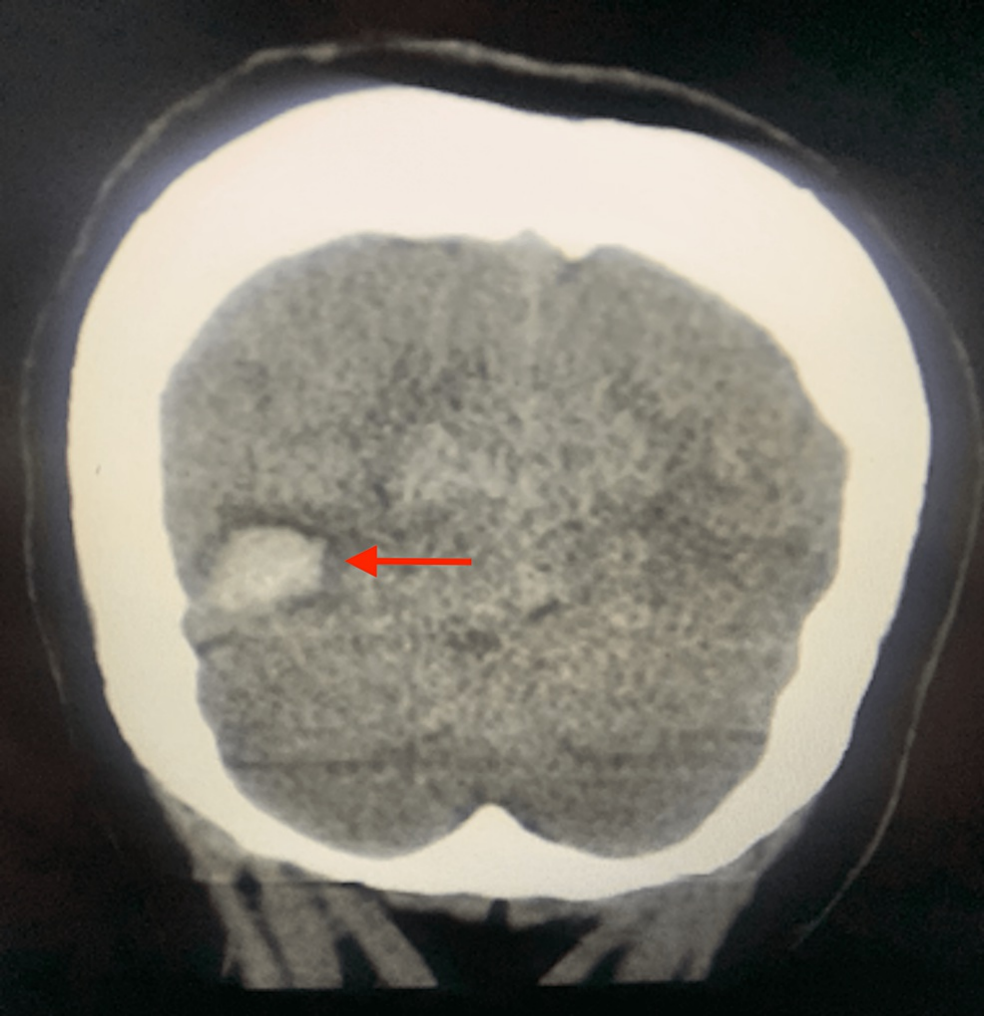
Coronal section from the initial brain CT without contrast done in the emergency department showing a right occipital hematoma

Initial vital signs were an oxygen saturation of 100% on room air, blood pressure 128/69, heart rate 68, and temperature 97.8 degrees Fahrenheit. Her Glasgow Coma Scale score was 15 and NIH Stroke Scale score was zero. A physical examination revealed an atraumatic, normocephalic head. A neurologic examination revealed cranial nerves II-XII to be intact, 5/5 strength and sensation intact in all extremities, intact visual fields, normal speech, and normal gait. Laboratory studies performed were within normal limits (Table 1). Computed tomography (CT) of the brain without contrast in the ED showed “acute appearing intraparenchymal hematoma in the right occipital lobe measuring 1.1 x 1.8 x 1.6 cm (3.2 mL)” (Figures 1, 2).

**Table 1:** Laboratory studies WBC: white blood cell, RBC: red blood cell, Hgb: hemoglobin, Hct: hematocrit, MCV: mean corpuscular volume, MCH: mean corpuscular hemoglobin, MCHC: mean corpuscular hemoglobin concentration, RDW: red cell distribution width, PT: prothrombin time, APTT: activated partial thromplastin time, INR: international normalized ratio, BUN: blood urea nitrogen

Test	Result	Reference range
WBC	7.5	3.8–11.0 10^3^/mm3
RBC	4.93	3.8–5.1 10^6^/µL
Hgb	15.0	12.0–16.0 g/dL
Hct	44.1	36%–46%
MCV	89.5	80–100 μm^3^
MCH	30.4	25–35 pg/cell
MCHC	34.0	31%–36% Hb/cell
Platelet count	321,000	150,000–400,000/mm^3^
PT	12.6	11–15 seconds
INR	1.12	0.9-1.2
APTT	33.1	25–40 seconds
Sodium	135	136–146 mEq/L
Potassium	4.8	3.5–5.0 mEq/L
Chloride	101	95–105 mEq/L
Carbon dioxide	21	21–34 mEq/L
BUN	16	7–18 mg/dL
Creatinine	0.6	0.6–1.2 mg/dL
Glucose	109	70–100 mg/dL
Calcium	9.5	8.4–10.2 mg/dL

Initial management of this case included prompt consultation to Neurosurgery and Neurology departments. Recommendations included a systolic blood pressure goal of less than 140 and ICU admission for frequent neurologic examinations. During admission, the patient was followed by both Neurology and Neurosurgery teams who agreed symptoms were consistent with prosopagnosia secondary to intraparenchymal hemorrhage involving the right fusiform gyrus. MRI of the brain with and without contrast was completed on day 2 of admission, which showed a "stable right occipital intraparenchymal hematoma with surrounding vasogenic edema". She did not require surgical intervention at any point. The patient did have significant anxiety related to seeing distorted faces and was seen by Psychiatry on day 5 of admission, but ultimately it was deemed that the prosopagnosia was related to the intraparenchymal hemorrhage.

Following discharge from the hospital, the patient had three outpatient follow-up appointments with the Neurosurgery department. Her first appointment was three and a half weeks after discharge and she had MR angiography (MRA) done showing no vascular malformations. At this appointment, the patient reported some improvement in facial recognition; however, faces of family members still appeared slightly distorted. At her second follow-up appointment approximately six weeks following discharge, she had a repeat MRI scan done that showed resolving intraparenchymal hematoma; symptoms still had not completely resolved, but continued to improve. At her third follow-up appointment approximately eight weeks following discharge, prosopagnosia had completely resolved and she had no residual deficits.

## Discussion

On arrival to the emergency department, this patient’s only deficit from her intracranial hemorrhage was difficulty with facial recognition. She did not have any cranial nerve deficits nor any visual field defects. A full neurological examination typical to an emergency department visit may not have any deficits in a case of acutely acquired prosopagnosia.

Acquired cases of prosopagnosia typically result from lesions in either the right fusiform face area (located on the inferior surface of the occipitotemporal cortex) or in the anterior temporal lobe (the amygdala or hippocampus) [1]. One etiology known as the “amnesia or associative variant” is caused by a lesion localized in the amygdala or hippocampal regions of the anterior temporal lobe. The amygdala makes various types of memories, and therefore, affected patients would develop difficulty remembering and recognizing other items besides faces [3]. To our knowledge, the amnestic or associative variant of prosopagnosia has not been documented as the only symptom in the setting of an acute insult, as the blood supply of the amygdala typically has three sources and is primarily supplied by an arteriole off the proximal middle cerebral artery [4].

Acquired prosopagnosia can be the only presenting symptom from an insult involving the right fusiform face area, which is supplied by branches off the right posterior cerebral artery (PCA) [5]. PCA strokes can often present with just one small neurologic complaint, such as prosopagnosia. Headache, paresthesia, visual agnosia (inability to recognize objects), alexia without agraphia (inability to read with the spared ability to write), achromatopsia (inability to recognize and/or see color), diplopia, visual field defects, and behavioral changes have also all been documented as singular complaints in the context of a new-onset PCA stroke [5]. These small neurologic findings may be the only symptom available to an emergency department physician in the context of an acute cerebral infarction, making definitive diagnosis elusive. They cannot be attributed to psychiatric or metabolic causes without assessment for possible intracranial pathology.

Posterior cerebral artery strokes, and other types of posterior circulation strokes, are often difficult to diagnose due to the variety of presenting symptoms that are easily attributed to other causes. Posterior strokes are about 2.5 times more likely to be missed compared to anterior circulation strokes, with one study showing initial misdiagnosis of 37% of posterior circulation strokes versus 16% of anterior circulation strokes [6]. Simply put, a posterior circulation stroke should be kept high on the differential in a patient with prosopagnosia.

## Conclusions

Prosopagnosia is a rare presentation of intracranial hemorrhage in the emergency department. Patients experiencing acquired prosopagnosia from intracranial hemorrhage can have a normal neurologic examination and a normal NIH Stroke Scale score, which can lead to a missed stroke diagnosis. A differential diagnosis in patients presenting with prosopagnosia should include intracranial hemorrhage, ischemic stroke, traumatic brain injury, neoplastic causes, infectious causes, and neurodegenerative disorders. Initial management in this particular case focused on blood pressure control, ICU admission, and appropriate consultations to Neurosurgery and Neurology departments; our patient's symptoms resolved spontaneously approximately eight weeks following discharge from the hospital. Currently, there are no clear evidence-based guidelines for the management of a patient presenting with prosopagnosia. However, a multidisciplinary team should be consulted and work together to provide the best care and quality of life for a patient with prosopagnosia.

## References

[1] Rocha Cabrero F, De Jesus O (2023). Prosopagnosia. StatPearls [Internet].

[2] Barton JJ, Corrow SL (2016). The problem of being bad at faces. Neuropsychologia.

[3] Gainotti G (2013). Is the right anterior temporal variant of prosopagnosia a form of 'associative prosopagnosia' or a form of 'multimodal person recognition disorder'?. Neuropsychol Rev.

[4] Goetzen B, Sztamska E (1999). Arterial vascularization of the amygdaloid nucleus in man and in sheep. (Article in French). Morphologie.

[5] Kuybu O, Tadi P, Dossani RH (2023). Posterior cerebral artery stroke. StatPearls [Internet].

[6] Arch AE, Weisman DC, Coca S, Nystrom KV, Wira CR III, Schindler JL (2016). Missed ischemic stroke diagnosis in the emergency department by emergency medicine and neurology services. Stroke.

